# Acute transverse myelitis caused by *Paragonimus* infection: a case report and review of the literature

**DOI:** 10.3389/fmed.2025.1506201

**Published:** 2025-05-08

**Authors:** Zhiguo Wen, Meiqiu Chen, Cong Xia, Yawei Sun, Yifan Zhang

**Affiliations:** ^1^School of Clinical Medicine, Guizhou Medical University, Guiyang, Guizhou, China; ^2^Department of Neurology, Affiliated Hospital of Guizhou Medical University, Guiyang, Guizhou, China

**Keywords:** *Paragonimus heterotremus*, myelitis, transverse, metagenomics, clinical features, diagnosis

## Abstract

Acute transverse myelitis (ATM) is a rare but severe neurological disorder that can be triggered by infections, autoimmune conditions, or other factors. While the association between parasitic infections and ATM is uncommon, *Paragonimus* infection can cause significant neurological damage, posing a diagnostic challenge. We report the case of a 65-year-old male patient who developed acute limb weakness, sensory loss, and fever following abdominal pain. Initial diagnostic tests were inconclusive; however, advanced imaging and metagenomic sequencing of cerebrospinal fluid ultimately confirmed an *Paragonimus* infection. After treatment with praziquantel, the patient successfully regained substantial motor function. This case highlights the need to consider parasitic infections in endemic areas and demonstrates the critical role of advanced diagnostic tools, such as metagenomic sequencing, in achieving timely diagnosis and treatment, ultimately improving the patient’s prognosis.

## Introduction

*Paragonimus* infection is a uncommon and complex parasitic disease with nonspecific clinical symptoms that vary widely, including hemoptysis, diarrhea, body pain, and limb weakness. This symptom diversity often leads to misdiagnosis. Neuroparagonimiasis, a severe complication of *Paragonimus* infection, presents with a wide range of neurological symptoms depending on the parasite’s location within the central nervous system (CNS). When the brain is affected, clinical manifestations may include meningitis, encephalitis, seizures, headaches, and visual disturbances. Although spinal cord involvement is less common, it can lead to acute transverse myelitis (ATM), characterized by sensory, motor, and autonomic dysfunction (1). In recent years, metagenomic sequencing has emerged as a key tool for diagnosing infectious diseases, particularly in identifying difficult-to-detect pathogens. This article provides a detailed discussion on the clinical features, diagnostic challenges, and treatment strategies of acute transverse myelitis (ATM) caused by *Paragonimus* infection, along with a clinical case. Our goal is to provide a reference for clinicians in the diagnosis and management of similar cases.

*Paragonimus* infection is a foodborne parasitic disease caused by a helminths parasite, with symptoms that are diverse and often unclear, leading to misdiagnosis. Infection occurs through the ingestion of raw or undercooked freshwater crustaceans (e.g., crabs or crayfish) contaminated with *Paragonimus* metacercariae. Once inside the host, the larvae penetrate the intestinal wall and typically migrate to the lungs. However, in rare cases, they may reach the central nervous system (CNS) via hematogenous dissemination or direct tissue penetration, resulting in neuroparagonimiasis. Research indicates that *Paragonimus* larvae can migrate from thoracic lesions to the epidural space of the spinal cord through the intervertebral foramina. This behavior aligns with the parasite’s characteristic migratory patterns, as it possesses the ability to penetrate the intestinal wall, pleura, and meninges (2). Although central nervous system involvement is relatively rare, it can cause serious complications such as acute transverse myelitis (ATM). Accurate diagnosis of such cases usually requires a combination of advanced diagnostic techniques. To date, no cases of acute myelitis caused by *Paragonimus* have been reported in China. Chest CT revealed bilateral pleural effusion, consolidation in the left lower lobe, and multiple mucus plugs in the trachea. Through diagnostic tests such as spinal MRI and cerebrospinal fluid analysis, he was diagnosed with *Paragonimus* infection. In addition to myelitis, the patient was also diagnosed with severe pneumonia, fungal infection, and cytomegalovirus infection. After multidisciplinary collaborative treatment, the patient’s condition was successfully managed.

This case highlights the importance of early identification of parasitic diseases in endemic areas, the significance of comprehensive multidisciplinary diagnostic and treatment approaches, and the critical value of detailed epidemiological history and etiology examination. The article also reviews relevant literature to enhance clinicians’ understanding of this disease.

## Case report

A 65-year-old married male was admitted with a 10-day history of fever, chest pain, abdominal pain, and 1 week of bilateral lower limb weakness. His symptoms began with a high fever reaching 39°C, accompanied by sharp pain in the bilateral subcostal and abdominal areas. Abdominal ultrasound performed at the local county hospital showed no abnormalities. After symptomatic treatment, including antibiotics, the patient’s condition worsened 3 days later, with the development of chest and back pain and progressively worsening lower limb weakness, making walking difficult. Eventually, he experienced sensory loss in both lower limbs, pain in his right hand and right side of the neck, occasional chest tightness, and shortness of breath. After 4 days of treatment, the patient’s vital signs stabilized, but his symptoms did not improve. He was transferred to the Affiliated Hospital of Guizhou Medical University for further evaluation on October 16, 2024. Upon arrival, he had not passed stool for 3 days, had a catheter in place, and was eating normally. Neurological examination: The patient was conscious but had slurred speech. The left corner of his mouth was slightly drooping, and both pharyngeal reflexes were absent. Neck movement was restricted, with pain in the right chest muscle upon tilting to the right side. Muscle strength in the right upper limb was grade 4, left upper limb strength was grade 5, and both lower limbs had no muscle strength (grade 0). Sensation below the umbilicus was reduced. Deep tendon reflexes were increased in both upper limbs (+++), but absent in both lower limbs. Muscle tone was decreased. Sensory loss was present in both lower limbs. The patient exhibited uncoordinated and imprecise finger-to-nose and rapid alternating movements of both upper limbs. No abnormalities were found on the remaining neurological examination.

### Laboratory and imaging findings

**Figure 1:**
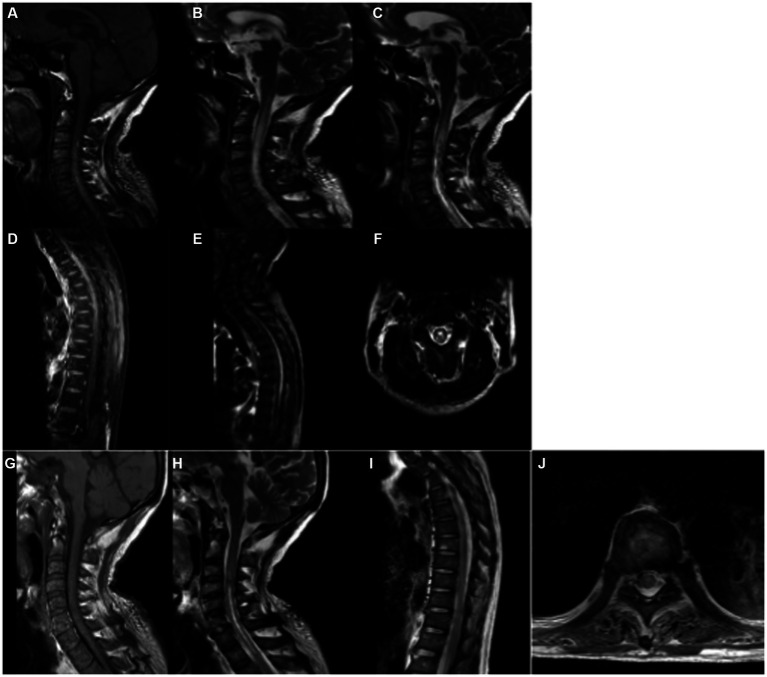
MR Imaging of *Paragonimus* Infection in the Spinal Cord of the Patient. Sagittal T1-weighted imaging (T1WI) **(A)** and T2-weighted imaging (T2WI) **(B)** show long segment abnormal signals parallel to the C1-T1 spinal cord levels. On T1WI, the signals are mostly isointense, while on T2WI, they appear hyperintense. Axial T2WI **(F)** reveals a high-intensity “figure-eight” sign, with a slightly lower signal ring-shaped cyst wall and a central area of high-signal necrotic tissue. Sagittal T1WI **(D)** and T2WI **(E)** demonstrate abnormal enhancement along the T1-T10 spinal cord levels, showing strip-like low-intensity signals within the area of abnormal enhancement. After 20 days of treatment, a follow-up MRI of the cervical and thoracic spine showed that in panels **(G–J)**, the inflammatory edema signals in the spinal cord had reduced or disappeared to varying degrees.

Initial blood tests showed neutrophils (87.30%) and lymphocytes (6.20%), with normal red blood cell count and hemoglobin levels. Coagulation and liver function tests were mostly unremarkable, though C-reactive protein was mildly elevated. Autoimmune antibody tests showed positive results for anti-Ro-52 and anti-mitochondrial antibodies (AMA-M2). Cerebrospinal fluid (CSF) analysis showed elevated white blood cell count (72 × 106/L), with 95% mononuclear cells, elevated protein levels (1,011 mg/L), and normal glucose and chloride levels. Interleukin-6 levels in the CSF were significantly elevated at 721.3 pg./mL. Second-generation sequencing of the CSF identified *Paragonimus* infection. Serum parasitic antibody testing confirmed positive results for *Paragonimus* IgG. MRI of the cervical and thoracic spine revealed lesions extending from C1 to T1 and from T1 to T10, consistent with spinal cord involvement (Figure 1).

### Additional findings

Chest CT revealed bilateral pleural effusion, consolidation in the left lower lobe, and multiple mucus plugs in the trachea. Sputum culture identified *Klebsiella pneumoniae*, *Aspergillus* fumigatus, and human cytomegalovirus. The patient’s medical history indicated that he had been living in the mountainous regions of Guizhou Province for an extended period, frequently consuming untreated raw water and raw fish, placing him at high risk for parasitic infections.

Based on the clinical presentation and diagnostic results, the patient was diagnosed with parasitic infection (*Paragonimus*), severe pneumonia, respiratory failure, acute transverse myelitis, and cytomegalovirus infection. Multidisciplinary treatment was initiated, and the patient’s condition gradually improved.

### Treatment and outcome

Before and after the definitive diagnosis, the patient began receiving treatment with methylprednisolone sodium succinate (500 mg, gradually tapered from October 17, 2023, to December 1, 2023) and immunoglobulin (22.5 g from October 17 to October 21, 2023, followed by small doses of 5 g maintained). During this period, the patient experienced vomiting, abdominal distension, high fever, and dyspnea. Blood gas analysis indicated Type I respiratory failure, leading to the implementation of tracheostomy and mechanical ventilation. Symptomatic treatment was provided based on multidisciplinary consultations. Once *Paragonimus* infection was confirmed, praziquantel treatment was initiated (first course from October 23 to October 25, 2023; second course from November 3 to November 8, 2023; third course from November 22 to November 27, 2023), with a dose of 1.2 g every 8 h. Additional treatments included antifungal therapy, neuroprotective treatment, antiviral therapy, blood pressure control, and bronchoscopy for sputum aspiration and drainage. Upon discharge, the patient’s vital signs were stable, and various indicators had improved. Neurological examination showed that the patient was alert, with speech intact. Muscle strength was 5/5 in both upper limbs, 1/5 distally and 0/5 proximally in both lower limbs. Sensation was reduced below the umbilicus. Reflexes were 3+ in the upper limbs, with absent lower limb reflexes and decreased sensation in both lower limbs. The patient was discharged in stable condition and continued rehabilitation treatment. Three months after follow-up, the patient’s symptoms had improved, and muscle strength in the lower limbs had recovered from 0/5 to 3/5.

## Discussion

According to the literature, spinal cord involvement in *Paragonimiasis* typically presents with a chronic or subacute onset. For instance, Oh (3) reported cases with chronic neurological symptoms, while Kusner and King (4) described similar prolonged disease courses, with patients frequently experiencing progressive lower limb weakness or sensory abnormalities. In contrast, the present case exhibited an acute onset, marked by fever and rapid progression to paralysis of both lower limbs. This presentation starkly differs from the typical descriptions in the literature, highlighting the diverse clinical manifestations of spinal cord involvement in *Paragonimiasis*. Regarding diagnosis, traditional approaches primarily rely on serological testing and MRI. However, these methods often suffer from limited specificity and sensitivity, leading to delays in diagnosis [Kusner and King (4)]. In comparison, the current case employed metagenomic sequencing of cerebrospinal fluid, which rapidly and definitively identified *Paragonimus* as the causative pathogen. This underscores the efficiency and precision of metagenomic sequencing as a novel diagnostic tool.

Clinical Significance: This case emphasizes the importance of promptly identifying symptoms and conducting a comprehensive assessment of the patient’s history, especially when diagnosing rare infectious myelitis. For patients living in endemic areas, a detailed epidemiological history can significantly enhance the detection of parasitic infections, particularly *Paragonimus* infection. Conventional diagnostic methods are often insufficient for atypical or rare infections, and clinicians must remain highly vigilant, particularly when spinal cord symptoms present with nonspecific features (5).

In this case, the combination of imaging and Next-Generation Sequencing (NGS) played a crucial role. Imaging helped identify the lesion’s location and characteristic signals (such as associated hemorrhagic degeneration) and provided tracking of lesion migration, thereby supporting the diagnostic decision (2, 6). NGS, with its high-throughput genomic sequencing, enabled the identification of pathogens, bypassing the limitations of traditional methods and significantly improving the accuracy and timeliness of the diagnosis (7, 8). Although NGS greatly enhances the early diagnostic efficiency for rare pathogens, particularly in cases of atypical or unclear infectious diseases, we must still consider how such cases could be diagnosed in the absence of these advanced tools.

In the absence of modern technologies, imaging studies still provide crucial clues to help confirm the location and nature of spinal cord lesions (6). Clinical symptoms (e.g., parasitic infections typically migrating from the gastrointestinal tract to the lungs and potentially spreading to the spinal cord) and the patient’s epidemiological history are essential for the initial diagnosis. Typical symptoms of parasitic infections include migratory abdominal pain, chest pain, and associated neurological dysfunctions (such as limb weakness and sensory disturbances), all of which suggest a possible spinal cord infection. Additionally, cerebrospinal fluid (CSF) analysis and serological tests (e.g., detecting antibodies for lung flukes) are valuable traditional diagnostic methods. Although modern techniques like NGS expedite the diagnostic process, traditional methods remain effective, particularly in resource-limited hospital settings.

Furthermore, this case further validates the importance of multidisciplinary collaboration and personalized treatment strategies in complex cases. The team promptly administered antiparasitic medications, such as praziquantel, adjusting the dosage and treatment course according to the patient’s condition. At the same time, corticosteroids and immunoglobulins were used to reduce spinal inflammation, effectively controlling the disease and preventing further neurological damage (9, 10). Multidisciplinary cooperation ensured control of fungal, bacterial, and viral infections, preventing multiple organ failure and leading to the patient’s eventual successful recovery.

## Conclusion

This case provides valuable insights into the diagnosis of rare infectious myelitis, highlighting the tremendous potential of combining next-generation sequencing (NGS) with imaging techniques. It also emphasizes the importance of multidisciplinary collaboration in the management of complex cases. The case further demonstrates the decisive role of early diagnosis, precise treatment, and collaborative care in improving the prognosis of rare diseases.

## Data Availability

The datasets used and/or analyzed during the current study are available from the corresponding author on reasonable request. All relevant clinical data have been anonymized to protect patient privacy.
